# Surgical Management of a Rare Inguinal Intestinal-Cutaneous Fistula with an Incarcerated Richter’s Femoral Hernia: A Case Report

**DOI:** 10.70352/scrj.cr.25-0327

**Published:** 2025-10-21

**Authors:** Saho Aso, Fuyuki Inagaki, Fuminori Mihara, Kenta Aso, Mai Nakamura, Takashi Kokudo, Norihiro Kokudo

**Affiliations:** 1 Department of Surgery, Hepato-Biliary Pancreatic Surgery Division, National Center for Global Health and Medicine, Tokyo, Japan; 2 Department of Hepatobiliary Pancreatic Surgery, Juntendo University School of Medicine, Tokyo, Japan

**Keywords:** inguinal intestinal-cutaneous fistula, Richter’s hernia, femoral hernia, incarcerated hernia

## Abstract

**INTRODUCTION:**

Richter’s hernia is a rare type of hernia in which only a part of the intestinal wall becomes entrapped, often leading to ischemia and necrosis. In rare cases, it can result in spontaneous formation of an intestinal-cutaneous fistula. Herein, we report a rare case of an intestinal-cutaneous fistula caused by incarceration of a Richter’s femoral hernia. Additionally, we present a brief literature review to highlight the diagnostic and therapeutic challenges associated with this condition.

**CASE PRESENTATION:**

An 81-year-old male with severe dementia presented with fecal leakage from the right groin. Physical examination revealed a 5-mm skin defect with stool discharge, and contrast-enhanced CT confirmed a small bowel skin fistula secondary to an incarcerated Richter’s femoral hernia. Given the patient’s stable condition and absence of peritoneal signs, initial conservative management was chosen. However, surgical intervention was performed because there was no improvement. Due to difficulty in dissection, an intraperitoneal approach was required. The affected bowel was resected, a functional end-to-end anastomosis was performed, and the hernial orifice was closed using a combined approach. The patient recovered uneventfully and was discharged.

**CONCLUSIONS:**

Prompt recognition and appropriate management are essential to improving outcomes in cases of Richter’s hernia complicated by intestinal-cutaneous fistula formation in the aging population.

## Abbreviation

PCR
: polymerase chain reaction

## INTRODUCTION

Richter’s hernia is characterized by entrapment of a portion of the intestinal wall, specifically the antimesenteric segment, within a narrow hernial orifice. Although this type of hernia commonly involves the distal ileum, it can occur at any site along the gastrointestinal tract, from the stomach to the colon.^1)^ Richter’s hernia occurs more frequently in the femoral ring; however, it can occur in the inguinal canal (12%–36%) and an incisional hernia (4%–25%).^2,3)^

The risk of intestinal necrosis in Richter’s hernia is significantly high owing to several factors. Entrapment within the hernial orifice applies intense pressure on the intestinal wall, and the segment opposite the mesentery is particularly prone to ischemia because of its rich terminal arteriole supply. Additionally, the partial nature of a hernia seldom causes luminal obstruction, resulting in minimal or no classical signs of intestinal obstruction. This atypical clinical presentation often contributes to diagnostic delays.^4)^ Among these complications, formation of an intestinal-cutaneous fistula secondary to intestinal perforation is a rare and severe manifestation of Richter’s hernia. Herein, we report a case of an intestinal-cutaneous fistula caused by incarceration of a Richter’s femoral hernia, highlighting the diagnostic and therapeutic challenges associated with this condition.

## CASE PRESENTATION

An 81-year-old male patient presented to our hospital with complaints of fecal leakage and groin pain. The patient reported a 1-week history of swelling in the right groin, with the onset of stool discharge from the swollen area a day prior to admission. The patient had no history of abdominal pain, constipation, nausea, or bowel disturbances. Past medical history included uveitis and severe dementia, with no history of prior surgical intervention.

**Fig. 1:**
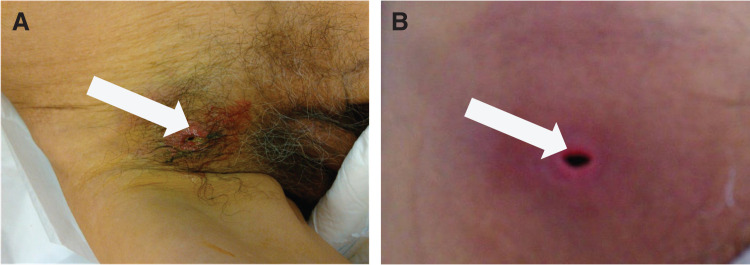
Physical findings (**A**) Physical findings at the time of the first visit. A 5-mm skin defect was observed in the groin area, accompanied by fecal leakage and surrounding redness (arrow). (**B**) An enlarged view of the cleaned skin defect (arrow).

**Fig. 2:**
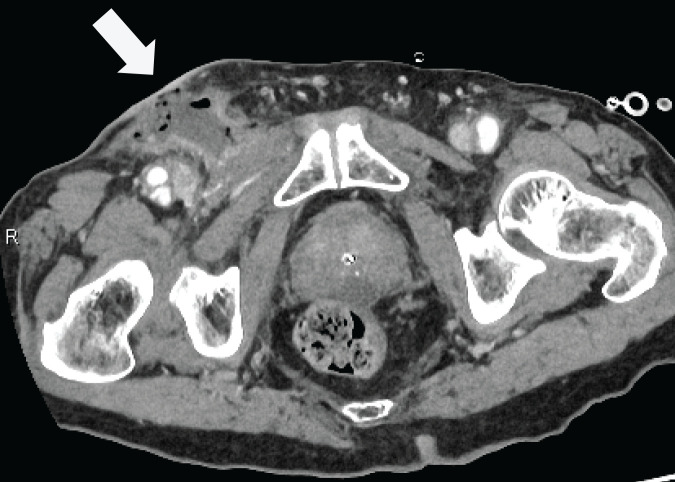
Preoperative contrast-enhanced CT imaging showing a small bowel hernia in the right groin. The intestinal wall within the hernia sac appeared indistinct, accompanied by increased subcutaneous fat tissue density and the formation of a skin fistula (arrow).

On presentation, his vital signs were stable, and the patient was afebrile. Physical examination revealed swelling, warmth, erythema, and a 5-mm skin defect in the groin area, with evident stool leakage (**Fig. 1A** and **1B**). Laboratory tests showed a mild decrease in albumin of 2.2 g/dL, and an elevated C-reactive protein level of 5.27 mg/dL, with no other abnormalities. Contrast-enhanced CT of the abdomen and pelvis demonstrated partial contrast effect loss in the small bowel wall, increased fat tissue density, and small bowel protrusion through the medial aspect of the femoral vein communicating with the skin surface (**Fig. 2**). Based on these findings, a small bowel-skin fistula secondary to a femoral hernia was suspected.

Given the absence of abdominal symptoms, minimal systemic inflammatory response, and no evidence of peritonitis on CT, emergency surgical intervention was deferred. Although the patient had no adverse respiratory symptoms, the findings of a routine COVID-19 polymerase chain reaction (PCR) test performed at admission proved positive. In view of the patient’s advanced age, a heightened risk of respiratory deterioration, and the considerable strain placed on medical resources during the pandemic, we considered emergency surgery in a COVID-positive patient as being associated with a significant perioperative risk. As the patient’s general condition was stable, conservative management was initiated with the intention of proceeding with surgery at a time when his condition and infectious status had become more favorable. However, given that there was no evident improvement in response to the conservative treatment, and that more than 7 days had passed since the positive COVID-19 PCR test, partial small bowel resection and inguinal hernia repair were performed 8 days after admission.

**Fig. 3:**
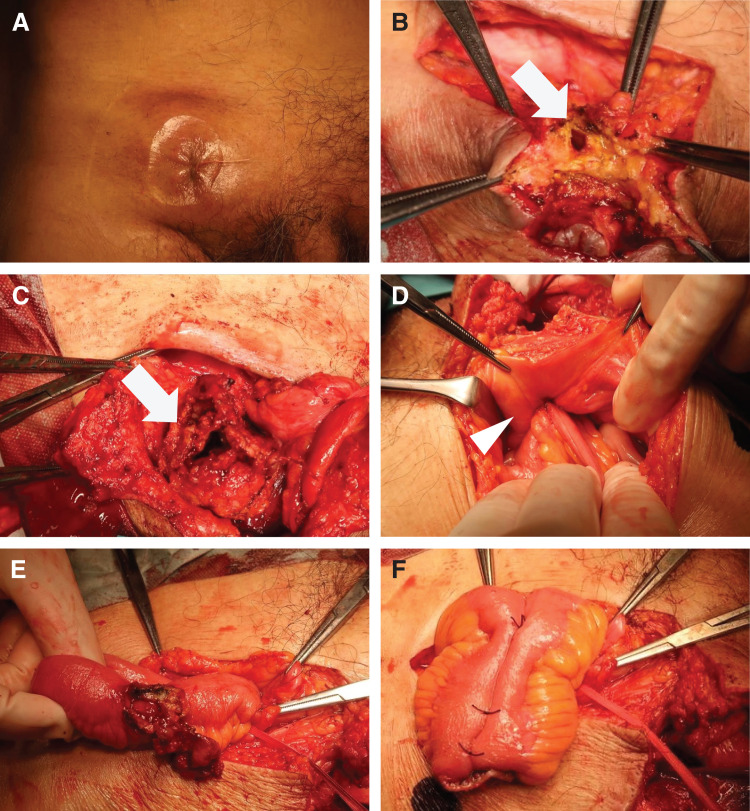
Intraoperative findings (**A**) The skin fistula is closed with skin sutures in advance. (**B**) The intestinal-cutaneous fistula originating from the femoral ring was identified (arrow). (**C**) Dissection from the surrounding tissue was performed, and the full extent of the intestinal-cutaneous fistula was exposed (arrow). (**D**) Inspection from the intraperitoneal side confirmed that it was a Richter's hernia with the small intestinal wall incarcerated in the femoral ring (arrowhead). (**E**) The incarcerated small intestine was released. (**F**) The portion of the intestinal-cutaneous fistula was resected and reconstructed using a functional end-to-end anastomosis.

The fistula was closed in advance using skin sutures (**Fig. 3A**). A 7-cm lateral incision was made at the midpoint between the right pubic symphysis and the right superior iliac spine. Although an external inguinal ring was identified, no intestinal prolapse was noted, confirming the diagnosis of femoral hernia. The skin surrounding the cutaneous fistula was incised, and the incision was extended to connect with the inguinal skin incision and wound to facilitate the identification of the hernial orifice (**Fig. 3B** and **3C**). However, dissection around the hernia orifice proved challenging, necessitating transition to an intraperitoneal approach. The herniated intestinal segment was transected distal to the hernial orifice (**Fig. 3D**). A midline lower abdominal incision was made to access the hernia orifice from the abdominal cavity. The affected intestinal segment was resected at the site of fistula formation, and a functional end-to-end anastomosis was performed to restore intestinal continuity (**Fig. 3E** and **3F**). Before closure, the surgical field was thoroughly irrigated. The hernial orifice was closed using a combined approach from both the abdominal and inguinal sides, with meticulous debridement of all necrotic tissue and suturing, whenever possible, only to healthy, viable tissue.

**Fig. 4:**
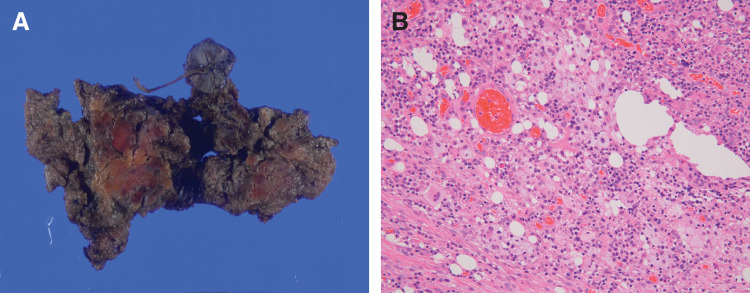
Pathological findings (**A**) Macroscopic findings revealed an intestinal-skin fistula and surrounding fibrous tissue. (**B**) Histopathological findings revealed congestion, angiogenesis, and prominent neutrophil infiltration in the granulation tissue, with inflammation extending from the adipose tissue to the dermis.

Histopathological examination revealed significant congestion, angiogenesis, and neutrophilic infiltration in the deep layers of the abdominal wall, consistent with severe inflammation (**Fig. 4A** and **4B**). The final diagnosis was an incarcerated Richter’s femoral hernia, classified as F-2 according to the Japanese Hernia Society Classification, with an associated intestinal-cutaneous fistula. Postoperative recovery was uneventful, and the patient was discharged on POD 11.

## DISCUSSION

Richter’s hernia is a type of hernia in which only one side of the intestinal wall becomes entrapped in the hernial orifice. It is characterized by rapid progression to gangrene.^4)^ Although Richter’s hernia is rare, the increasing adoption of minimally invasive surgical techniques has led to multiple reports of Richter’s hernia at laparoscopic port sites, highlighting the importance of understanding its management.^5–7)^ In rare cases, when surgical intervention is delayed, spontaneous drainage of intestinal contents may occur, resulting in intestinal-cutaneous fistula.^4)^

An intestinal-cutaneous fistula is an abnormal communication between the gastrointestinal tract and skin, frequently associated with complications such as sepsis, electrolyte imbalance, skin excoriation, wound dehiscence, and malnutrition.^8,9)^ Approximately 75%–85% of intestinal-cutaneous fistulas are iatrogenic, whereas 15%–25% occur spontaneously, often secondary to conditions such as inflammatory bowel disease, malignant tumors, and diverticulitis.^8,10,11)^

However, reports in English language specifically describing intestinal-cutaneous fistulas caused by incarcerated Richter’s hernia remain limited. We conducted a systematic literature search using the PubMed database on May 18, 2025, to identify published reports. A combination of the following search terms was used: (“Richter’s hernia” OR “Richters hernia” OR “partial enterocele”) AND (“intestinal cutaneous fistula” OR “bowel skin fistula” OR “enterocutaneous fistulas” OR “fistula”) AND (english[Filter]). The literature search identified nine case reports in English language, excluding iatrogenic cases. The characteristics of these cases, along with the present case, are summarized in **Table 1**.^1,12–19)^

**Table 1:** Summary of Reported Cases of Intestinal-cutaneous Fistula Resulting From Hernia

Reference	Year	Age/Sex	Type of hernia	Side	Resected area	Strangulation	Surgery	Postoperative complications
Habib Faridi et al.^1)^	2013	55/M	Inguinal hernia	Left	Terminal ileum	No	Laparotomy small bowel resection + Ileostomy	None
Weledji et al.^12)^	2014	70/F	Femoral hernia	Left	Ileum	No	Laparotomy small bowel resection + Hernia repair	Death (POD 12)
Ahi et al.^13)^	2015	62/M	Inguinal hernia	Right	Terminal ileum	No	Laparotomy small bowel resection + Hernia repair	None
Elenwo et al.^14)^	2016	61/F	Inguino-labial hernia	Left	Ileum	Yes	Laparotomy small bowel resection + Hernia repair	Wound infection and breakdown
Chen et al.^15)^	2017	62/F	Intervertebral disk hernia	Right	Terminal ileum	No	Laparotomy small bowel resection + Hernia repair	None
Hajong et al.^16)^	2017	53/M	Inguinal hernia	Left	Jejunum	No	Laparotomy small bowel resection	None
Talukder et al.^17)^	2017	60/M	Inguinal hernia	Right	Ileum	Yes	Laparotomy small bowel resection + Debridement	Death (POD 5)
Zia et al.^18)^	2017	76/F	Femoral hernia	Left	Ileum	Yes	Laparotomy small bowel resection + Hernia repair + Ileostomy	N/D
Baig et al.^19)^	2022	60/M	Umbilical hernia	Left	Transverse colon	No	Laparoscopic transverse colectomy + Hernia repair	None
Present case	2023	81/M	Femoral hernia	Right	Ileum	No	Laparotomy small bowel resection + Hernia repair	None

F, female; M, male; N/D, no data

Mean age of the patients was 62 years. Hernia types included inguinal hernia (four cases), femoral hernia (two cases), inguinolabial hernia (one case), umbilical hernia (one case), and intervertebral disk hernia (one case). The hernias were left-sided in six cases and right-sided in three. The incarceration site was predominantly the ileum, as observed in the present case. The primary surgical approach was laparotomy; however, laparoscopic procedures have also been recently reported. Owing to the high risk of infection, hernia repair using a mesh was avoided in all the cases, and direct suture repair was performed in some cases. There were two cases of postoperative mortality. In one patient, death was attributed to an underlying condition of acquired immunodeficiency syndrome. In the other case, the patient presented 3 days after the onset of fecal leakage from the fistula and succumbed to sepsis.

Surgery in older adults is generally considered high-risk, and emergency laparotomies involving bowel resection in patients aged ≥80 years with an American Society of Anesthesiologists classification of ≥3 are associated with a high perioperative mortality rate.^20)^ The risk factors for mortality following enterocutaneous fistula formation include malnutrition, sepsis, and electrolyte imbalances.^21,22)^ The modified Frail Index-5, comprising five elements—the presence of diabetes, hypertension, congestive heart failure, chronic obstructive pulmonary disease, and functional independence—has been reported as a useful tool for predicting prognosis.^23)^ The patient had no comorbidities other than severe dementia, and he was independent during activities of daily living. Although mild hypoalbuminemia was observed, this may reflect inflammation rather than malnutrition. Sepsis or electrolyte imbalance was not observed. Additionally, the spontaneous formation of the intestinal-cutaneous fistula allowed external drainage of fecal matter, preventing intra-abdominal contamination, which may have contributed to the patient’s favorable overall condition. These combined factors are thought to have resulted in the favorable outcomes observed in this patient.

In the present case, the patient was older than the previously reported average age. Although swelling was noted in the right groin, the patient did not recognize the formation of a skin fistula even after approximately 1 week, partly due to the dementia. Richter’s hernia is often minimally symptomatic; in older adults or cognitively impaired patients, it may progress over time to the formation of a skin fistula without recognition. As the population ages, the number of similar cases is also likely to increase. In the present case, the patient’s general condition was stable, resulting in favorable outcomes. However, given the potential for fatal outcomes, timely and appropriate management is crucial.

## CONCLUSIONS

We encountered a case of intestinal-cutaneous fistula caused by an incarcerated Richter’s femoral hernia. With an aging population, the incidence of similar cases is likely to increase, highlighting the need for appropriate and timely management.

## DECLARATIONS

### Availability of data and materials

The datasets supporting the conclusions of this study are included in this article and its additional files.

## References

[1] Habib Faridi S, Siddiqui B, Amanullah Khan M, et al. Suprapubic fecal fistula due to Richter’s inguinal hernia: a case report and review of literature. Iran J Med Sci 2013; 38: 129–31. 23825893 PMC3700059

[2] Frankau C. Strangulated hernia: a review of 1487 cases. Br J Surg 1931; 19: 176–91.

[3] Gillespie RW, Glas WW, Mertz GH, et al. Richter’s hernia: its etiology, recognition, and management. AMA Arch Surg 1956; 73: 590–4. 13361710

[4] Steinke W, Zellweger R. Richter’s hernia and Sir Frederick Treves: an original clinical experience, review, and historical overview. Ann Surg 2000; 232: 710–8. 11066144 10.1097/00000658-200011000-00014 PMC1421226

[5] Romanelli JR, Roshek TB 3rd, Lynn DC, et al. Single-port laparoscopic cholecystectomy: initial experience. Surg Endosc 2010; 24: 1374–9. 20039073 10.1007/s00464-009-0781-z

[6] Khuroo S, Wani AA, Kaur I, et al. Unusual Richter’s hernia: impacted foreign body leading to incarceration and perforation-A rare clinical entity. Int J Surg Case Rep 2021; 79: 492–5. 33757269 10.1016/j.ijscr.2021.01.088 PMC7889443

[7] Rammohan A, Naidu R. Laparoscopic port site Richter’s hernia-an important lesson learnt. Int J Surg Case Rep 2011; 2: 9–11. 22096675 10.1016/j.ijscr.2010.11.002 PMC3199732

[8] Whelan JF Jr, Ivatury R. Enterocutaneous fistulas: an overview. Eur J Trauma Emerg Surg 2011; 37: 251–8. 26815107 10.1007/s00068-011-0097-2

[9] Gribovskaja-Rupp I, Kosinski L, Ludwig KA. Obesity and colorectal cancer. Clin Colon Rectal Surg 2011; 24: 229–43. 23204938 10.1055/s-0031-1295686 PMC3311490

[10] Berry SM, Fischer JE. Classification and pathophysiology of enterocutaneous fistulas. Surg Clin North Am 1996; 76: 1009–18. 8841361 10.1016/s0039-6109(05)70495-3

[11] Hollington P, Mawdsley J, Lim W, et al. An 11-year experience of enterocutaneous fistula. Br J Surg 2004; 91: 1646–51. 15505866 10.1002/bjs.4788

[12] Weledji EP, Puepi MA, Chichom AM. A rare spontaneous enterocutaneous fistula. J Surg Case Rep 2014; 2014: rju121. 25391523 10.1093/jscr/rju121 PMC4228200

[13] Ahi KS, Moudgil A, Aggarwal K, et al. A rare case of spontaneous inguinal faecal fistula as a complication of incarcerated Richter’s hernia with brief review of literature. BMC Surg 2015; 15: 67. 26018618 10.1186/s12893-015-0055-8 PMC4446841

[14] Elenwo SN, Igwe P, Jamabo R, et al. Spontaneous entero-labial fistula complicating Richters hernia: Report of a case. Int J Surg Case Rep 2016; 20: 27–9. 26785080 10.1016/j.ijscr.2016.01.003 PMC4818293

[15] Chen W, Liu L, Huang H, et al. A case report of spontaneous umbilical enterocutaneous fistula resulting from an incarcerated Richter’s hernia, with a brief literature review. BMC Surg 2017; 17: 15. 28193213 10.1186/s12893-017-0216-z PMC5307766

[16] Hajong R, Khongwar D, Komut O, et al. Spontaneous enterocutaneous fistula resulting from Richter’s Hernia. J Clin Diagn Res 2017; 11: PD05–06. 10.7860/JCDR/2017/27789.10370 PMC5620839 28969198

[17] Talukder S, Gupta A, Singh BN, et al. Fistulating Richter’s hernia of groin with necrotizing soft tissue infection: a lethal combination. J Clin Diagn Res 2017; 11: PD05–07. 10.7860/JCDR/2017/28201.10195 PMC5583875 28892969

[18] Zia MK, Anwar H, Naz S, et al. Richter’S Hernia Presenting As Faecal Fistula In Female; A Rare Entity. J Ayub Med Coll Abbottabad 2017; 29: 493–5. 29076691

[19] Baig SJ, Priya P. Complexities in the management of a Richter’s supraumbilical hernia with colocutaneous fistula in a patient with morbid obesity: a case report with a review of literature. J Minim Access Surg 2022; 18: 308–10. 35313440 10.4103/jmas.JMAS_99_21 PMC8973488

[20] Hajibandeh S, Hajibandeh S, Hughes I, et al. Development and validation of HAS (Hajibandeh index, ASA status, sarcopenia)-a novel model for predicting mortality after emergency laparotomy. Ann Surg 2024; 279: 501–9. 37139796 10.1097/SLA.0000000000005897

[21] Martinez JL, Luque-de-León E, Ballinas-Oseguera G, et al. Factors predictive of recurrence and mortality after surgical repair of enterocutaneous fistula. J Gastrointest Surg 2012; 16: 156–63; discussion 163-4. 22002412 10.1007/s11605-011-1703-7

[22] Ravindran P, Ansari N, Young C, et al. Definitive surgical closure of enterocutaneous fistula: outcome and factors predictive of increased postoperative morbidity. Colorectal Dis 2014; 16: 209–18. 24521276 10.1111/codi.12473

[23] Alser O, Naar L, Christensen MA, et al. Preoperative frailty predicts postoperative outcomes in intestinal-cutaneous fistula repair. Surgery 2021; 169: 1199–205. 33408040 10.1016/j.surg.2020.11.018

